# Oral mucosa: an alternative epidermic cell source to develop autologous dermal-epidermal substitutes from diabetic subjects

**DOI:** 10.1590/1678-77572016-0217

**Published:** 2017

**Authors:** Daniela GUZMÁN-URIBE, Keila Neri ALVARADO-ESTRADA, Mauricio PIERDANT-PÉREZ, Bertha TORRES-ÁLVAREZ, Jesus Martin SÁNCHEZ-AGUILAR, Raúl ROSALES-IBÁÑEZ

**Affiliations:** 1 Universidad Autónoma de San Luis Potosí, Facultad de Estomatología, Grupo de Investigación en Ingeniería Tisular, San Luis Potosí, México.; 2 Universidad Autónoma de San Luis Potosí, Facultad de Medicina, Maestría en Ciencias en Investigación Clínica, San Luis Potosí, México.; 3 Hospital Central Dr Ignacio Morones Prieto, Departamento de Dermatología, San Luis Potosí, México.; 4 Universidad Nacional Autónoma de México, Facultad de Estudios Superiores Iztacala, Laboratorio en Ingeniería Tisular y Medicina Traslacional, Tlanepantla, México.

**Keywords:** Oral mucosa, Skin substitutes, Biological dressings

## Abstract

Oral mucosa has been highlighted as a suitable source of epidermal cells due to its intrinsic characteristics such as its higher proliferation rate and its obtainability. Diabetic ulcers have a worldwide prevalence that is variable (1%-11%), meanwhile treatment of this has been proven ineffective. Tissue-engineered skin plays an important role in wound care focusing on strategies such autologous dermal-epidermal substitutes.

**Objective:**

The aim of this study was to obtain autologous dermal-epidermal skin substitutes from oral mucosa from diabetic subjects as a first step towards a possible clinical application for cases of diabetic foot.

**Material and Methods:**

Oral mucosa was obtained from diabetic and healthy subjects (n=20 *per* group). Epidermal cells were isolated and cultured using autologous fibrin to develop dermal-epidermal *in vitro* substitutes by the air-liquid technique with autologous human serum as a supplement media. Substitutes were immunocharacterized with collagen IV and cytokeratin 5-14 as specific markers. A Student´s t- test was performed to assess the differences between both groups.

**Results:**

It was possible to isolate epidermal cells from the oral mucosa of diabetic and healthy subjects and develop autologous dermal-epidermal skin substitutes using autologous serum as a supplement. Differences in the expression of specific markers were observed and the cytokeratin 5-14 expression was lower in the diabetic substitutes, and the collagen IV expression was higher in the diabetic substitutes when compared with the healthy group, showing a significant difference.

**Conclusion:**

Cells from oral mucosa could be an alternative and less invasive source for skin substitutes and wound healing. A difference in collagen production of diabetic cells suggests diabetic substitutes could improve diabetic wound healing. More research is needed to determine the crosstalk between components of these skin substitutes and damaged tissues.

## Introduction

Advancements in the field of tissue engineering have allowed the widespread use of skin substitutes to cover wounds and to promote healing^19^. Great progress has been achieved in the culturing of keratinocytes and fibroblasts; this has been especially true for keratinocytes since the first successful culture by Rheinwald and Green in 1975^25^, to the present day, with the creation of specialized *in vitro* techniques^14,22,23,24^. Although current tissue-engineered skin substitutes have shown promising results for wound healing^6,8,11,13,26^, there are still several challenges to overcome, therefore there is a continuing search for novel approaches to wound care and treatments that could be more effective, where all epidermal cells and matrices are obtained from the same patient, with less invasive sources to obtain the biological components, and with new alternatives to harvest tissue in patients with physiological and ethical limitations due to their conditions.

Oral mucosa has been highlighted as a viable alternative source of epidermal cells, due to its easy preparation and suitable features, such as higher cell proliferation rates, lower terminal cell differentiation degrees and an increased biological activity compared with epidermal keratinocytes^22^. This tissue also has the advantage that its harvesting produces less disability, and provides better aesthetic outcomes. With these special features, it is assumed that the skin substitute obtained from oral mucosa can be produced faster. A possible disadvantage for the use of oral mucosa as an epidermal cell source could be the differentiation of the epithelial pattern of keratinocytes from oral mucosa, which differs from that of epidermal keratinocytes and skin. The skin is an example of an orthokeratinized epithelium. It displays a stratum basale, stratum spinosum, stratum granulosum and stratum corneum. Gingiva tissue also consists of an epithelium on a connective tissue matrix populated mainly with fibroblasts and endothelial cells, however, in contrast to skin, the epithelium is parakeratinized^27^. In this regard, Ueda, et al.^26^ (1995) reported in their study that a grafted sheet of keratinocytes from oral mucosa changed the epithelial pattern over a period of 4 weeks, explaining this phenomenon due to epithelial-mesenchymal interactions, suggesting that the specificity of the epithelium is dependent on the influence of the underlying mesenchymal tissue.

Worldwide, diabetic foot is a major medical, social and economic problem. It is estimated that approximately 15% of the more than 150 million people with diabetes world-wide will at some stage develop diabetic foot ulceration^7^. Foot problems are indeed a global problem and there is no area in the world that does not report the development of foot lesions as a consequence, mainly of neuropathy and peripheral vascular disease. The prevalence of active foot ulceration varies from approximately 1% in certain European and North American studies to more than 11% in reports from some African countries^7^. Although there have been many developments in recent years, which encourages optimism for future improvement in diabetic foot care, there is still much to be done.

The aim of the present study was to obtain an autologous dermal-epidermal skin substitute using oral mucosa tissue from diabetic subjects with a measurable immunofluorescence characterization, as a first step to perfecting the development technique with the main focus of future possible clinical applications on the treatment of diabetic foot ulcers. Also it proposes for the first time, maintaining this substitute using autologous serum, thus avoiding the use of animal products and promoting the development of the construct.

## Material and methods

This was an experimental *in vitro* test carried out at Hospital Central “Dr. Ignacio Morones Prieto”, the Basic Sciences Laboratory, Faculty of Stomatology, Universidad Autónoma de San Luis Potosí. The study was performed in accordance with the Helsinki Declaration, and it was approved by the Faculty’s Ethical Board (CEIFE-033-012).

### Subjects

Twenty adult subjects with diabetes mellitus type II (DM2), diagnosed according to the American Diabetics Association guidelines^16^, and 20 healthy controls donated 20 ml of peripheral blood and a sample of oral mucosa from the retromolar area. Blood was collected in labeled Vacutainer tubes, 10 ml without additives, and 10 ml with sodium citrate (BD *Vacutainer* ^®^, Franklin Lakes, NJ, USA) and frozen at 20°C, until used. Oral mucosa samples were obtained with a 3 mm biopsy punch (Miltex, Davies Drive York, PA, USA) previous aseptic and antiseptic controls and inferior alveolar nerve blockage (IAN). Hemorrhage was controlled by direct means for 10 minutes. Patients received instructions for wound care, and a follow-up after 7 days. None of the patients presented complications.

### Isolation and cell culture

Each oral mucosa sample was collected in 1.5 ml tubes containing phosphate buffered saline (PBS), 100 μg/ml streptomycin, 100 IU/ml penicillin, and 10 μg/ml amphotericin B (all from Sigma-Aldrich, St. Louis, MO, USA) and preserved at 4°C for 12 h. Oral mucosa was macroscopically separated by region, with a scalpel, into epithelium and connective tissue. By using the explant technique, each region was grown separately in 25 cm^2^ culture dishes (Nunc, Roskilde, Denmark). DMEM low glucose medium (Sigma-Aldrich, St. Louis, MO,USA) supplemented with 10% fetal calf serum (Sigma-Aldrich, St. Louis, MO, USA) was used as culture medium for fibroblasts. Keratinocytes were cultured using QN medium (a specific culture medium for keratinocytes). After 3 days the fetal calf serum concentration was reduced to 5%, and after a further 3 days to 1%. Blood samples were defrosted and centrifuged at 1200 rpm for 10 minutes. Serum and plasma were isolated and kept at -20°C until use. Autolougus serum was used for cell culture instead of the fetal calf serum after 7 days, while the plasma was used to develop the stroma for the substitutes. Both cell lines were incubated at 37°C in an atmosphere of 5% CO_2_ until the cultures reached a confluence of 80%. The culture media was changed every third day.

### Cell cultures characterization

Once the cultures reached a confluence of 80%, part of the cell cultures were used for an indirect immunofluorescence assessment. In order to assure that the cells were fibroblasts and keratinocytes, the characterization of these cell cultures was performed. The specific monoclonal antibody used for fibroblasts was anti-collagen I (Santa Cruz Biotechnology, Paso Robles, CA, USA) mainly because due to its abundance in the skin, recognized as the most abundant collagen in the epidermis, and its specificity for this cell type. For keratinocyte was anti-cytokeratins 5-14 (Santa Cruz Biotechnology, Paso Robles, CA, USA). As secondary antibodies, Alexa fluor 488 (Anti-Rabbit IgG polyclonal, Invitrogen, Waltham, MA, USA) and Alexa 594 (Anti-mouse monoclonal IgG, Invitrogen, Waltham, MA, USA) were also used. Briefly, cells were grown on round glass cover slips (12 mm). After 12 h of incubation, the cultures were fixed for 20 minutes with neutral formalin, permeabilized with Triton X100 0.025% for 20 min at room temperature then blocked with bovine serum albumina (BSA) 1% for 45 min. The primary antibodies were incubated for 2 h at room temperature and the secondary antibodies were incubated for 2 h at room temperature and were protected from light. Between each step, phosphate buffered saline rinses were made. The samples were observed by confocal microscopy (Leica, Model DMI4000B, Wetzlar,Germany) and LASAF^®^ software (Leica, Wetzlar, Germany).

### Development of the dermo-epidermal substitute

#### Stromal phase

**Figure 1:**
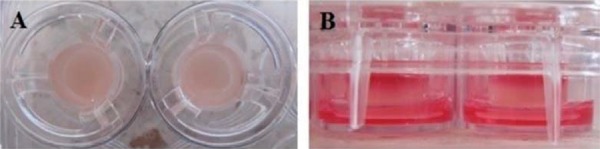
A. Air-liquid technique phase 1: Development of stroma with the fibrin/agarose gels. B. Specific culture medium placed in a Transwell System in accordance with the air-liquid technique

Fibroblasts were detached from culture dishes using TrypLE Express (Invitrogen, Waltham, MA, USA), centrifuged and suspended once more in the medium and quantified by One Scepter (Millipore, Billerica, MA, USA). Cells were again suspended in DMEM low glucose medium (Sigma-Aldrich, St. Louis, MO, USA) with 1% of human serum obtained from each subject, reaching a cell concentration of 500,000 *per* 1 mL. A solution was made by mixing the resuspended cells with plasma and agarose at 3% to create fibrin agarose/gels^5^. 300 microliters of the solution were placed in the Transwell system (Corning, Midland, NC, USA) following the air-liquid technique^3^. Culture medium was placed covering the entire substitute. Each system was incubated at 37°C in a 5% CO_2_ atmosphere and with a relative humidity of 95% for 10 days (Figure 1a, b).

**Figure 2:**
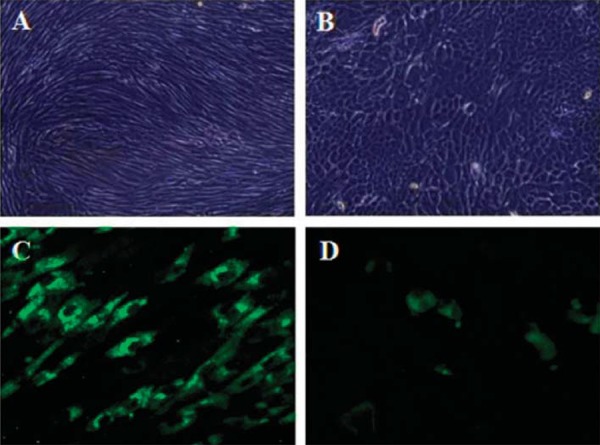
A. Fibroblast culture at confluence. B. Keratinocyte culture at confluence. C. Positive immunofluorescence for fibroblasts (anti-collagen-I). D. Positive immunofluorescence for keratinocytes (anti-cytokeratin 5-14)

#### Epithelial phase

Cultured keratinocytes were detached from the culture dishes using TrypLE Express (Invitrogen, Waltham, MA, USA), centrifuged and resuspended in a QN medium with 1% human serum obtained from, and quantified by One Scepter (Millipore, Billerica, MA, USA) to reach a concentration of approximately 500,000 cells *per* 1 mL. One hundred microliters of the cell solution were placed in the Transwell system (Corning, Midland, NC, USA) on the top of the already set stroma, and incubated for 10 days using the air-liquid technique. Culture medium was placed covering both compartments on the first day of settlement, on the fourth day, the culture medium was placed only in the lower compartment. Each system was incubated at 37°C in a 5% CO_2_ atmosphere with a 95% relative humidity for 10 further days.

## Dermal-epidermal substitute evaluation and characterization

Substitutes were mechanically separated from each system and placed in dishes with 1X PBS to remove excess of the medium. Subsequently they were fixed with 4% paraformaldehyde and placed in a solution of 3% sucrose as a cryoprotector. Cryosections of 6 microns thickness were made at -29°C in a cryostat (Leica Model CM1510S-3, Wetzlar, Germany). From each sample, 100 cuts were made and placed on 50 slides (two cuts *per* slide). Each slide was treated for its indirect immunofluorescence assessment.

For the characterization of these dermal-epidermal substitutes, the specific monoclonal antibodies used were anti-collagen IV and anticytokeratins 5-14 (Santa Cruz Biotechnology, Paso Robles, CA, USA). Collagen IV was assessed in this characterization due to its role as the primary collagen found in the extracellular basal membranes separating a variety of epithelial and endothelial cells. It is a major component of the dermal-epidermal junction, where it is mostly found in the lamina densa of the basal membrane^9^. The basal membrane zone mediates tissue compartmentalization and sends signals to epithelial cells about the external microenvironment, and also has important structural and functional effects on blood vessels, constituting an extracellular microenvironment sensor for endothelial cells and pericytes^9^. Meanwhile, Cytokeratins 5-14 (CK5-14) are expressed on basal keratinocytes and the expression of these filaments are characteristic of complex stratified epithelia^1^. Keratin 14 (CK14) is a prototypic marker of dividing basal keratinocytes and helps in the maintenance of epidermal cell shape; it also provides resistance to mechanical stress. Interestingly, the CK5/CK14 pair is expressed in the basal layer of the epidermis, which contains epidermal stem cells and transient amplifying (TA) cells^2^. Monoclonal secondary antibodies, Alexa fluor 488 and 594 (Invitrogen, Waltham, MA, USA) were also used. Ten different fields were chosen randomly from each slide; each slide of every substitute created to be evaluated, was chosen by the sample function of the *R program,* version 3.0.1. The evaluation of the slide was performed by an observer blinded to the nature of the study. A total of 20 images were obtained; observations were performed by confocal microscopy (Leica, Model DMI4000B, Wetzlar, Germany) and the LASAF^®^ software (Leica, Wetzlar, Germany). Images obtained were evaluated by Image J program manager (1.46a version, NIH) with the ROI function getting the mean of the arbitrary fluorescence units *per* slide, *per* antibody for further statistical analysis.

## Cell growth curve

Cell growth curves were performed (N=8). Cultures of 4 healthy subjects and 4 age-matched diabetic subjects were selected. Fibroblasts were subculture in duplicate in 6 well boxes by placing 100,000 cells *per* well. The evaluation times were 0, 3, 6 and 9 days.

## Statistics

Statistical analysis at 95% of confidence was performed using JMP 8 software (SAS Institute Inc., Cary, NC, USA) and R 3.1.3^20^. An analysis of descriptive statistics, obtaining the measures of a central tendency and dispersion of all the variables was then performed. For the bivariate comparative analysis we used the Student’s t-test for continuous variables based on a normal distribution calculated with plot of Fox^10^. For categorical variables the Chi-squared test was applied. A paired Student´s t test for time 0 versus 3, 6 and 9 days for the growing cellularity between the groups was performed. Statistical significance was considered with a p value <0.05. For the cell growth curve, statistical significance was considered with a Bonferroni correction of alpha for 3 comparisons with a p value of <0.016.

## Results

### Study demographics

A total of 40 oral mucosa samples were collected (n=20 *per* group) according to the previously set criteria. Of the total of samples taken, 19 corresponded to men and 21 to women. The average age was 52.15±13.5 years. Variables studied in each group are showed in Table 1. Characteristics including age and sex were relatively homogenous between both groups.

**Table 1:** Studied variables *per* group

	DIABETIC SUBJECTS GROUP (N=20)	HEALTHY SUBJECTS GROUP (N=20)	P VALUE
Age	57.55*±11.59†	47*±15.92†	0.75‡
Sex (M/F)	9/11	10/10	0.02§
Culture days	25.3*±3.09†	26.85*±1.78†	0.05‡
AFU Col-IV	8.05*±1.59†	6.15*±3.16†	0.02‡
AFU Ck 5-14	9.56*±3.741†	10.47*±5.86†	0.05‡
AFU Total	17.61*±5.22†	16.62*±8.94†	0.67‡

*Mean

†Standard deviation

‡ Student´s t-test

§Chi-Square

Match paired Student´s T-test

### Obtaining and characterization of cell cultures

Fibroblast and keratinocyte cultures were established from the oral mucosa from diabetic and healthy subjects (Figure 2a, b). Cell cultures achieved 80% percent of confluence at different times; diabetic cultures had a culture day average of 25.3±٣.٠٩, meanwhile the healthy subjects’ culture day average was 26.85±١.٧٨. Cell morphology showed by cultures of the two cell lines corresponded to the typical morphology of fibroblasts and keratinocytes. Fibroblast´ cultures were positive for anti-collagen I, and keratinocyte cultures were positive for anti-cytokeratins 5 and 14 (Figure 2c, d).

### Cell growth curve

**Figure 3:**
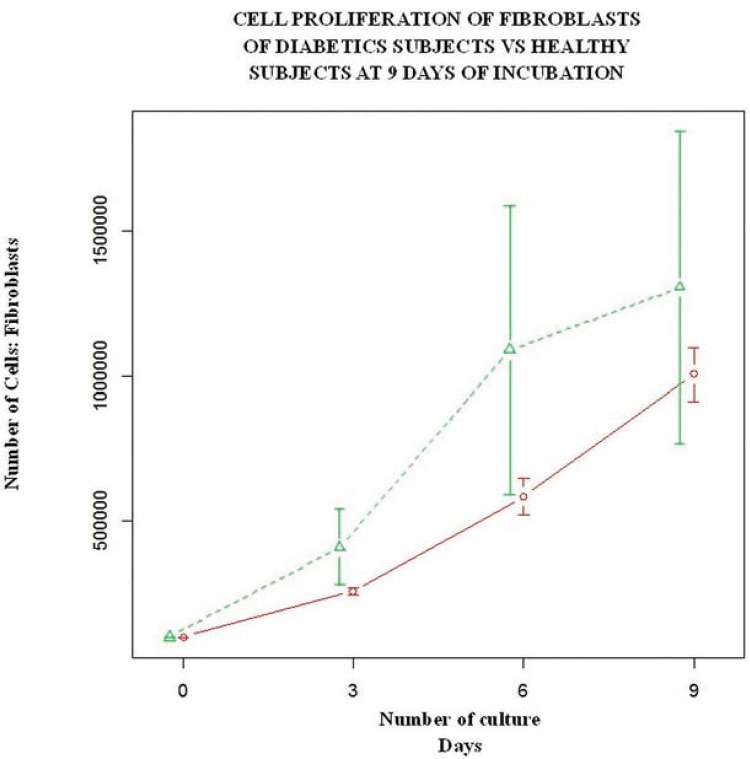
Cell growth curve. Cell proliferation of the fibroblasts of diabetics subjects vs healthy subjects after 9 days of incubation. Diabetic subjects (green) had an increased cell proliferation since day 3 in comparison with healthy subjects (red)

Cell proliferation results showed a significant increased growth in the diabetic group at time 0 vs 3 days (p=0.009), and time 0 vs 9 days (p=0.004); however statistically wide confidence intervals are showed, indicating greater variability in the behavior of the diabetic group compared with the healthy group (Figure 3, Table 2).

**Table 2:** Cell proliferation at the growth cell curve

	DIABETIC SUBJECTS GROUP (N=4)	HEALTHY SUBJECTS GROUP (N=4)	P VALUE
Basal	100.000	100.000	1‡
Day 3	409,999*±258,686†	257,500*±20615†	0.009||
Day 6	1’089,999*±991,407†	585,000*±123693†	0.02||
Day 9	1’305,000*±1’075,648†	1’005,000*±185022†	0.004||

*Mean

†Standard deviation

‡ Student´s t-test

§ Chi-Square

||Match paired Student´s T-test

### Development of the dermo-epidermal substitute

**Figure 4:**
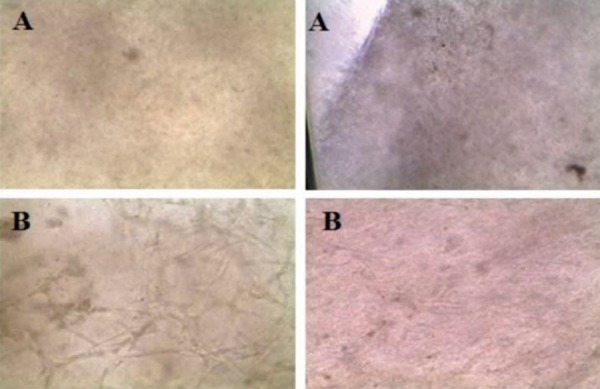
A. Dermal-epidermal substitutes of healthy subjects observed under the inverted microscope (40X). A globular pattern is observed. B. Dermal-epidermal substitutes of diabetic subjects observed under the inverted microscope (40X). A fibrillar pattern is observed

**Figure 5:**
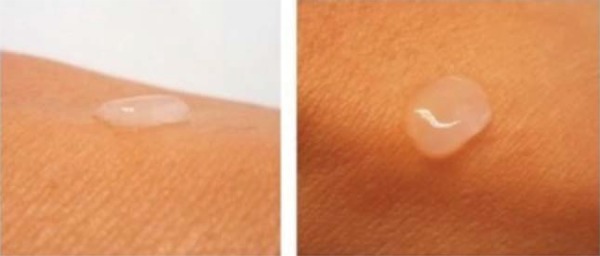
Clinical appearance of the developed dermal-epidermal skin substitute

Substitutes were developed using the air-liquid technique, as previously described. The culture time for the stromal and epithelial phase was 10 days for each. Once both phases were completed, diabetic and healthy substitutes were observed with an inverted microscope at 40X, showing interesting features. An extended cellular pattern with more fibers was observed in the diabetic substitutes, whereas healthy substitutes showed a globular pattern with fewer fibers (Figure 4 a, b). The clinical appearance of the substitutes was clear and humid with an adherence to surfaces (Figure 5).

### Dermal-epidermal substitute evaluation and characterization

**Figure 6:**
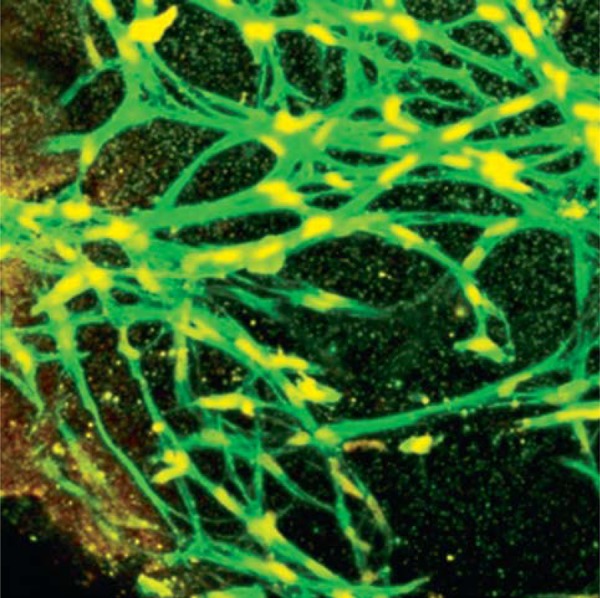
Dermal-epidermal skin substitute, immunostained, showing cell distribution. Red (Sytox, Invitrogen), green (Phalloidin, Invitrogen)

Each substitute was characterized and assessed as previously described. Anti-cytokeratin 5-14 expression showed 1.14 arbitrary fluorescence units (AFU) less in the diabetic substitutes compared with the healthy control group. Anti-collagen IV expression was 1.9 AFU higher in the diabetic substitutes when compared with the healthy group. In addition, dermo-epidermal skin substitutes were immunostained with Sytox Red (Invitrogen, Waltham, MA, USA) showing the nucleus and the cytoskeleton was stained with Phalloidin (Invitrogen, Waltham, MA, USA), showing the cell distribution throughout the thickness of the substitute (Figure 6).

### Statistical analysis

A Student’s T-test was performed as planned. All variables showed a normal distribution. There was a significant difference between the diabetic group and the healthy group for arbitrary fluorescence units of collagen IV (AFU col-IV) p value=0.02 and arbitrary fluorescence units of cytokeratin 5-14 (AFU ck 5-14) p value=0.05 (Table 1).

## Discussion

Skin tissue engineering is a therapeutic field developed to aid in the healing of injured skin, especially for wounds that have difficulty healing in the primary stage. This study showed the development of the dermo-epidermal substitutes of oral mucosa obtained from the retromolar area with a 3 mm punch biopsy from diabetic and healthy subjects, using the air-liquid technique and its assessment by immunofluorescence characterizing and quantifying its intensity in AFU, as a first step towards a possible future clinical application. Recent studies show oral mucosa to be a feasible, alternative source of epidermal cells and matrices, mainly for its intrinsic characteristics studied and described in previous studies. Iida, et al.^13^ (2005) developed an epidermal equivalent from oral mucosa based on an acellular allogeneic dermal matrix showing good results after its clinical application^13,26^. Other studies reported the development of skin substitutes using cells obtained from biopsies of human skin, mainly the foreskins of children or from established epidermal cell lines. The main disadvantage of this approach is the allogeneic nature of the cells and the latent possibility of short-term rejection by the host. Still, wound healing was accomplished with its clinical application^6,8,11^.

Primary cultures of fibroblasts and keratinocytes were achieved from both populations (diabetic and healthy subjects) by the explant technique. Eighty percent of confluence was achieved in 25.3 days for diabetic subjects and for healthy subjects in 26.85 days. In similar studies, the production of primary fibroblast and keratinocyte cultures has been reported by the explant technique alone^8^, and also using enzymatic techniques such as cell disruption without mentioning the time at which they achieved confluence, pointing out only the use of cells in different culture passages^11,13,24^. In primary cell culture techniques, cell disintegration is a strategy that can reduce culture times up to 50%, yet has the main disadvantage of the possibility of damaging tissues and cells^12^. It is important to point out that for the clinical application of these skin dermal-epidermal substitutes, cell culture times should be reduced, and thereby the enzymatic digestion technique is a good strategy to be used for future studies.

Regarding the development of dermo-epidermal substitutes, the air-liquid technique was an efficient cell culture technique for obtaining these skin equivalents. It provides the conditions and characteristics needed for the obtention of this kind of tissue, mainly by the gas exchange (CO_2_ and O_2_) of the epithelial layer in the final stage. It allows for a place specific culture medium for each cell line (fibroblasts and matrices in the lower compartment and keratinocytes in the upper compartment) throughout the entire culture process^20^. In our study, the construct remained intact throughout the *in vitro* process, allowing for the manipulation of both compartments independently compared with other culture techniques and materials^11,21,22,24^. Culture times reported through the air-liquid technique range from day 1 of the stromal stage, up to 5 days, and the epithelial phase for a period of 5 to 10 days^21^. In this study we used 10 days in the cultures for both phases; stromal and epithelial, obtaining good results in relation to the establishment of the stromal cells as evidenced by the immunofluorescence evaluation.

In the assessment of the substitutes by immunofluorescence, there was a significant difference observed between both groups (p=0.002 for AFU COL-IV and p=0.05 for AFU CK5-14). The results showed less arbitrary fluorescence units for keratinocytes (CK5-14 antibody) in the substitutes generated from the oral mucosa of diabetic subjects (9.56) compared to those generated from the oral mucosa of the healthy subjects (10.47). In relation to the above, recent studies show an association of Neurotensin (NT), a neurotransmitter involved in the process of wound healing. Moura, et al.^15^ (2014) studied the effects of neurotensin on human keratinocytes under hyperglycemic conditions and normal conditions at different functional levels, mainly NT receptors, cytokines and growth factor expression and proliferation and also migration of the epidermal cells. They observed that the neurotensin did not affect the viability of keratinocytes, however, neurotensin and the expression levels of all recipients were significantly reduced. Neurotensin treatment stimulated the expression of neurotensin receptor 2 (NTR2), whereas expression levels of neurotensin receptors 1 and 3 (NTR1, NTR3) did not change. Keratinocyte proliferation was not affected, but the migration of keratinocytes was reduced. Their results show that hyperglycemic conditions adversely affect the endogenous expression of neurotensin and its receptors, especially in keratinocytes NTR2 with important consequences for its function. This effect of neurotensin in hyperglycemic conditions has also been well studied in other cells such as macrophages, where it was observed that this neurotransmitter runs cell migration patterns and the inflammatory response in wound healing^18^. Given these findings, Moura, et al.^16,17^(2014) conducted two additional studies where neurotensin was added as a component to the dermo-epidermal substitutes, specifically for the scaffold or biomaterial in which cells are suspended. They tested in mediums of collagen and chitosan observing a reduction in inflammatory infiltrates up to day 3 after implantation in rats with induced diabetes, and also an improvement in wound healing promoting cell migration and deposition of collagens I and III^16,17^. Future studies concerning the effect of NT are required in order to assess if in these neurotensin substitutes have an inhibitory proliferation role.

Fibrosis is a complication of chronic hyperglycemia involving the excessive proliferation of extracellular matrix (ECM) and its accumulation in various tissues and organs, the most prominent being microcirculation, kidney, heart, retina and wound healing. Chronic hyperglycemia affects the cells responsible for the production of collagen IV causing this accumulation^4^. Our results support this statement, as it showed increased immunofluorescence for anticollagen-IV and fibroblasts of the dermal-epidermal substitutes generated from diabetic subjects (8.05) compared with healthy subjects (6.15). Berlanga-Acosta, et al.^5^(2010) reported that under the high glucose load imposed by diabetes, skin and skin fibroblasts were disturbed in recreated *in vitro* clinical models showing alterations to the normal physiology of fibroblasts as well as extracellular matrix secretion, therefore, it has been suggested that high concentrations of glucose is the major trigger of a cascade of molecular changes to skin fibroblasts. In our study, cell cultures of diabetic subjects reached an 80% confluence in a shorter time (25.3 days) than healthy subjects (26.85 days) suggesting a permanent effect of hyperglycemic conditions in cell physiology; however further study is needed.

Differences in the collagen production of diabetic subjects’ cells could suggest that diabetic substitutes might improve the healing of diabetic foot ulcers, however more research is needed to determine the clinical impact of the differences found. Additionally these substitutes may be used to test new drugs in an environment that is closer to that of real tissues.

Results of our study were measurable and quantifiable (intensity of collagen-IV and anticytokeratin 5-14 in arbitrary fluorescence units) showing numerical variations in the amount of expression of both antibodies in both populations; diabetic and healthy subjects, suggesting differences in cellular function under this clinical condition and, with possible implications in the clinical application of the substitute. Also it is necessary to emphasize that the cells not only survived through the development process of the substitute, but also they were integrated in to the dermal matrix and secreted basal membrane supported by the positive expression of the specific markers CK5-14 and COL IV.

Despite the above, the significance of these findings is not yet known, further studies are needed to elucidate them. However this data may provide a basis to perfect/improve the development technique of the dermal-epidermal substitutes from the oral mucosa of diabetic patients and to answer the biological questions raised by it.

Within the constraints of our study, it is worth mentioning that no *in vitro* tests were performed to evaluate the functionality of the developed substitute, such as migration essays. In that matter, the next step would be *in vivo* and *in vitro* essays of functionality of these substitutes developed by this technique and the characterization proposed in this study. This will lead us to the future clinical application of these diabetic skin substitutes.

## Conclusions

It is possible to develop dermal-epidermal substitutes from the isolation of the epidermal cells of oral mucosa from diabetic and healthy subjects using the air-liquid technique and its assessment by immunofluorescence characterizing and quantifying its intensity in arbitrary fluorescence units (AFU).

Dermal-epidermal skin substitutes of diabetic subjects showed reduced keratinocyte immunofluorescence intensity when compared to the substitutes from healthy subjects; while for fibroblasts and COLIV, substitutes of diabetic subjects showed a greater intensity, with a higher AFU value. These findings suggest differences in cellular function under this clinical condition; however more research is needed to determine the crosstalk which occurs between the components of these skin substitutes and the damaged tissues. The following long-term goal in this research is the clinical application of these substitutes; further *in vivo* animal studies to evaluate functionality will be performed to continue with this matter.
